# The Effect of Selected Nitric Oxide Synthase Polymorphisms on the Risk of Developing Diabetic Nephropathy

**DOI:** 10.3390/antiox13070838

**Published:** 2024-07-13

**Authors:** Magdalena Król-Kulikowska, Mirosław Banasik, Marta Kepinska

**Affiliations:** 1 Department of Pharmaceutical Biochemistry, Faculty of Pharmacy, Wroclaw Medical University, Borowska 211a, 50-556 Wroclaw, Poland; magdalena.krol-kulikowska@umw.edu.pl; 2 Department and Clinic of Nephrology and Transplantation Medicine, Faculty of Medicine, Wroclaw Medical University, Borowska 213, 50-556 Wroclaw, Poland; miroslaw.banasik@umw.edu.pl

**Keywords:** diabetes nephropathy, renal replacement therapy, single-nucleotide polymorphisms, NOS isoforms

## Abstract

Background: Nitric oxide synthase (NOS) is an enzyme that catalyzes the formation of nitric oxide (NO), the altered production of which is characteristic of diabetic nephropathy. NOS exists in three isoforms: NOS1, NOS2, and NOS3. Moreover, there are reports about the potential role of *NOS3* polymorphisms in the development of diabetes complications. The aim of this study was to assess the role of selected *NOS* polymorphisms—rs3782218 (*NOS1*), rs1137933 (*NOS2*), rs1799983, rs2070744, and rs61722009 (*NOS3*)—in the risk of developing diabetic nephropathy and in the likelihood of renal replacement therapy. Methods: The studied polymorphisms were analyzed in a group of 232 patients divided into three groups. Four polymorphisms (rs3782218, rs1137933, rs1799983, rs2070744) were genotyped using the PCR-RFLP, while the rs61722009 polymorphism was genotyped using the PCR. Results: The C/C genotype and the C allele of the rs3782218 polymorphism (*NOS1*) were associated with an increased risk of developing diabetic nephropathy and an increased likelihood of renal replacement therapy. In turn, the G allele of the rs1137933 polymorphism (*NOS2*) reduces the likelihood of renal replacement therapy. Conclusions: The specific genotypes or alleles of the rs3782218 (*NOS1*) and rs1137933 (*NOS2*) polymorphisms seem to be potential risk factors for diabetic nephropathy and renal replacement therapy.

## 1. Introduction

In modern medicine, diabetes is classified as a lifestyle that cannot only significantly reduce the quality of life but also lead to serious complications [1,2,3]. One of the most common complications of type 1 and type 2 diabetes is diabetic nephropathy, which often leads to the need for renal replacement therapy [4,5,6]. To understand these somewhat complicated relationships, it is necessary to refer to the pro-/antioxidant balance disorders accompanying diabetes and its complications. Hyperglycemia increases the production of free radicals and reactive oxygen species (ROS), thus causing oxidative stress [7,8]. However, despite studies indicating that increased oxidative stress causes the progression of diabetic nephropathy, the accurate mechanisms are still not fully understood [9]. Analogous compounds to ROS are reactive nitrogen species (RNS)—a group of molecules derived from nitric oxide (NO). RNS have unpaired electrons; thus, they are characterized by high chemical reactivity [10]. Their excessive production causes the phenomenon of nitrosative stress, which also accompanies many diseases, including diabetes and its complications [11].

NO is produced in a reaction catalyzed by nitric oxide synthase (NOS). There are three known isoforms of this enzyme—NOS1, NOS2, and NOS3—and each of them is encoded by a different gene [12]. According to the literature, early nephropathy occurring in diabetes is associated with increased intrarenal NO production, mainly mediated by neuronal nitric oxide synthase (NOS1, nNOS) and endothelial nitric oxide synthase (NOS3, eNOS) [13]. Increased NO production may contribute to hyperfiltration as well as microalbuminuria, which are characteristic of the early stage of diabetic nephropathy [14]. On the other hand, most studies indicate that advanced nephropathy leads to severe proteinuria and that the deteriorating condition is associated with hypertension accompanied by progressive NO deficiency. Several factors, including hyperglycemia, increased formation of advanced glycation end products, increased oxidative stress, and the activation of certain proteins, contribute to reducing NO production or its availability [15,16].

The expression of all three NOS isoforms can be detected in pancreatic β cells. NOS1 can be found in insulin secretory granules [17]. In turn, in insulin resistance, NOS2 participates in the deregulation of metabolic processes in tissues by disturbing glucose and lipid homeostasis and causing endothelial dysfunction through the local and systemic formation of an inflammatory environment [18]. This is due to increased nitrosative stress, which affects the activity of proteins that are involved in maintaining glucose metabolism [19]. Moreover, polymorphisms of genes encoding NOS isoforms may also play a role in NO-related abnormalities that contribute to the development and progression of insulin resistance, type 2 diabetes (T2D), and diabetic nephropathy [20,21,22,23]. 

Garme et al. [24] showed that the C/T genotype of the rs1137933 polymorphism in *NOS2* was associated with an increased risk of developing T2D. In addition, the T allele of this polymorphism was also associated with an increased risk of progression of this disease. Other studies also demonstrated associations between the rs1137933 polymorphism and increased susceptibility to the development of T2D, but no similar relationship was observed in patients with diabetic nephropathy [25]. However, this study was only performed on the Chinese Han population, so it should be extended to include other populations to exclude or confirm their influence on the results.

The *NOS3* polymorphisms seem to be equally important in the development of T2D and its complications. The variable number of tandem repeat (VNTR) polymorphisms in the *NOS3* has been shown to be associated with the development of diabetic nephropathy. A study of 598 patients has shown that it may be associated with the progression of diabetes mellitus and, consequently, diabetic nephropathy [26]. Another study, which concerned the assessment of *NOS3* polymorphisms in the context of the risk of developing diabetic nephropathy, concerned the analysis of rs1799983, rs2070744, and 27-bp VNTR polymorphisms [27]. The T allele of the rs1799983 polymorphism and the C allele of the rs2070744 polymorphism were significantly more frequent in patients with diabetic nephropathy than in patients without nephropathy. However, in the case of 27-bp VNTR polymorphism, no significant change in NO concentrations was found. The obtained results suggest that *NOS* polymorphisms may indeed be genetic determinants of diabetic nephropathy in patients with type 2 diabetes.

The aim of this study was to assess the frequency of selected polymorphisms of genes encoding all three NOS isoforms—NOS1, NOS2, and NOS3—in patients with diabetic nephropathy, both after and without kidney transplantation. The following polymorphisms were analyzed: rs3782218 (*NOS1*), rs1137933 (*NOS2*), rs1799983, rs2070744, and rs61722009 (*NOS3*). The rs3782218 polymorphism is mainly associated with an increased risk of cardiovascular diseases, where the T allele shows an additive protective effect against coronary heart disease (CHD) and hypertension [28]. However, due to the fact that both diabetes and its complications are often accompanied by this type of disease, it was decided to include it in these studies. This polymorphism is located in a regulatory region of *NOS1*. It belongs to single-nucleotide polymorphisms (SNPs) and consists of replacing cytosine with thymine [29]. The rs1137933 polymorphism also belongs to the SNP type of polymorphism; it is located in exon 10 of *NOS2* and consists of replacing cytosine with thymine. This is a missense mutation [25]. The rs1799983 and rs2070744 polymorphisms also belong to the SNP-type polymorphisms. The first one is located in exon 7 of *NOS3* and consists of replacing guanine with thymine. This is a missense mutation [30,31]. The second polymorphism is located in the promoter region of *NOS3* and consists of replacing cytosine with thymine [31]. The last polymorphism—rs61722009—is a VNTR-type polymorphism and is located in intron 4. It is often found under other names, such as 27-bp VNTR polymorphism or 4a/4b polymorphism [32].

Additionally, the relationship between specific genotypes of the above-mentioned polymorphisms and the concentration of individual NOS isoforms, creatinine, and C-reactive protein (CRP) in blood serum and glucose in blood plasma was also examined. The relationship between individual genotypes within the studied polymorphisms and the concentrations of zinc and copper was also taken into account, as these are elements that are of fundamental importance in the pathophysiology of diabetes and its complications [33,34].

## 2. Materials and Methods

### 2.1. Study Groups

The research material was blood samples obtained from 232 patients who participated in this study, divided into three groups: the control group (*N* = 50), the diabetic nephropathy group (*N* = 85), and the kidney transplant diabetic nephropathy group (*N* = 97). Consent of the Bioethics Committee at Wroclaw Medical University (no. KB 835/2021) was obtained for the use of the collected biological material for research purposes. All respondents were acquainted with research issues and gave their consent in writing to collect biological material. The sample size was determined by power analysis using preliminary data from previous studies, with assumptions of α = 0.05 and a power of 80%. These data were obtained as part of unpublished research conducted on a group of patients with type 2 diabetes, the aim of which was to check the impact of the rs1799983 polymorphism in *NOS3* on the risk of developing this disease. In order to determine the sample size, differences in allele frequencies (in the context of the rs1799983 polymorphism) and biochemical parameters (concentrations of NOS3, glucose, CRP, zinc, and copper) were used. Based on the results obtained, it was estimated that the recruited sample size was adequate to observe the expected differences.

Biological samples were obtained from Łukasiewicz PORT—Polish Center for Technology Development (control group)—and from the Department and Clinic of Nephrology and Transplantation Medicine of the Wroclaw Medical University (diabetic nephropathy group and kidney transplant diabetic nephropathy group). Blood was collected into tubes with clotting activator (to obtain serum; cat. No.: BD 368815, Becton Dickinson, Franklin Lakes, NJ, USA) and tubes with EDTA (to obtain plasma and buffy coat; cat. No.: BD 367864, Becton Dickinson, USA). DNA was isolated from the buffy coat using a ready-made isolation kit (Syngen Blood/Cell DNA Mini Kit, cat. No.: SY221012, Syngen Biotech, Wrocław, Poland).

The control group consisted of respondents with excluded cardiovascular diseases, liver function disorders (measured by gamma-glutamyltransferase—GGT; alanine aminotransferase—ALT; and aspartate aminotransferase—AST activity), atherosclerosis, diabetes (based on insulin and fasting glucose measurements), hypertension (blood pressure measurements), inflammation (C-reactive protein concentration), and tumors. The use of drugs or dietary supplements in the last 6 months was also an exclusion criterion.

The selection of diabetic nephropathy patients was made on the basis of medical history, laboratory tests, and imaging tests (e.g., USG). The following parameters were measured in patients: creatinine, blood morphology, urine general examination (including the presence of protein), albuminuria, sodium/potassium, glucose, and eGFR (estimated glomerular filtration rate; calculated according to the abbreviated formula MDRD—Modification of Diet in Renal Disease). Qualification for this study required the presence of diabetes and one of the following: albuminuria, proteinuria, or increased creatinine levels. The exclusion criterion was the presence of other causes of kidney damage. In addition, patients received a questionnaire in which we collected information such as age, gender, anthropometric data (weight, height), other chronic diseases, the use of stimulants (smoking cigarettes, alcohol consumption), or medications (Questionnaire S1, Supplementary Materials). In order to characterize the groups, the following parameters were used: age, sex, BMI values, glucose and creatinine concentrations, eGFR values, and CRP concentrations. The characteristics of the three studied groups have already been described in a previous study that focused on the role of *ACE* polymorphisms in assessing the risk of developing diabetic nephropathy [35].

### 2.2. Methods

#### 2.2.1. Determination of NOS1, NOS2, NOS3, Glucose, Creatinine, eGFR, and CRP Concentrations

Serum NOS1 concentration was measured using the Human Nitric Oxide Synthase, Brain, NOS1 ELISA Kit (cat. No.: E0924Hu, Bioassay Technology Laboratory, Shanghai, China). Serum NOS2 concentration was measured using the Human Nitric Oxide Synthase, Inducible, INOS-NOS2 ELISA Kit (cat. No.: E4710Hu, Bioassay Technology Laboratory, Shanghai, China). Serum NOS3 concentration was measured using the Human Endothelial Nitric Oxide Synthase (eNOS) ELISA Kit (cat. No.: MBS265088, MyBioSource, Inc., San Diego, CA, USA). Glucose, creatinine, and CRP concentrations were measured in the hospital laboratory during routine patient visits. eGFR values were calculated according to the abbreviated MDRD formula.

#### 2.2.2. Determination of Metal Concentrations

The metal concentrations (zinc—Zn; copper—Cu) in the blood serum were measured using the SOLAAR M6 atomic absorption spectrophotometer (Thermo Elemental Solaar House, Cambridge, UK) at the Department and Clinic of Internal and Occupational Diseases, Hypertension and Clinical Oncology, Wroclaw Medical University. To measure the concentrations of these metals, the flame atomic absorption spectrometry (FAAS) method was used in an air–acetylene flame.

#### 2.2.3. Genotyping Analysis

The four polymorphisms (rs3782218, rs1137933, rs1799983, rs2070744) were determined using the polymerase chain reaction and restriction fragment length polymorphism analysis (PCR-RFLP). In turn, the rs61722009 polymorphism (*NOS3*), due to the fact that it is the VNTR polymorphism, was determined using the polymerase chain reaction (PCR). Primers were designed with the Primer-BLAST program based on gene sequences from the GenBank (National Center for Biotechnology Information). The sequences of the primers, reaction conditions, and the restriction enzyme used in this study are presented in Table 1.

The digested DNA fragments were visualized using a 2% or 3% agarose gel with Green DNA Gel Stain (cat. No.: SY 521031, Syngen Biotech, Wrocław, Poland). Electropherograms showing restriction digest products are provided in Figures S1–S5 (Supplementary Materials).

#### 2.2.4. Statistical Analysis

Statistical analyses were performed using the STATISTICA 13.3 package (Statsoft Polska, Sp. z o.o., Kraków, Poland) under the Wroclaw Medical University license.

The frequencies of genotypes were compared using the χ^2^ test and Fisher’s exact test. In order to check whether all populations are in Hardy–Weinberg equilibrium, the χ^2^ test was performed.

Normality of distribution was checked using the Shapiro–Wilk test, and homogeneity of variances was checked using Levene’s test. To test statistical significance between groups, the nonparametric Kruskal–Wallis test with Dunn’s post hoc test was used.

Logistic regression analysis was performed to assess the significance of the effect of polymorphism genotypes on the risk of diabetic nephropathy and the likelihood of renal replacement therapy, expressed as odds ratios (OR) with a 95% confidence interval (CI). Statistical significance was considered for *p* < 0.05.

## 3. Results

### 3.1. Concentrations of the NOS Isoforms in the Studied Groups

Lower NOS1 and NOS2 concentrations were observed in the diabetic nephropathy group compared to the control group (*p* = 0.002, *p* < 0.001, respectively).

**Figure 1:**
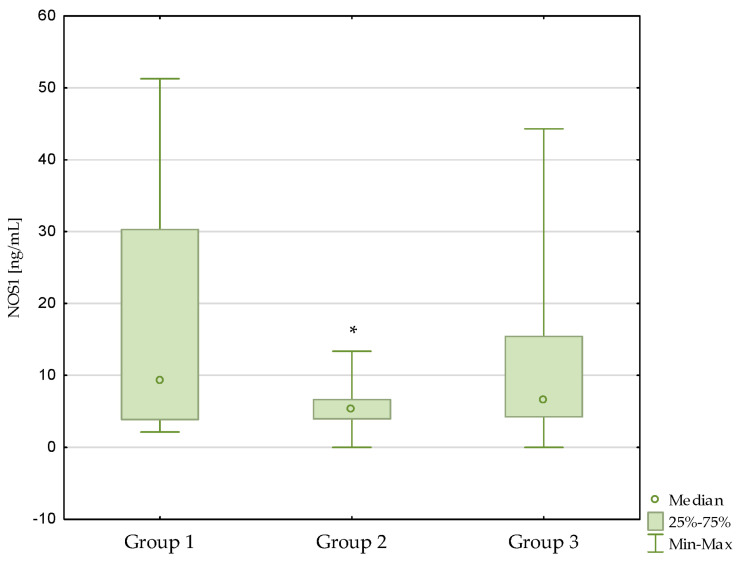
Concentrations of NOS1 in the studied groups. Group 1—control group; Group 2—diabetic nephropathy group; Group 3—kidney transplant diabetic nephropathy group. * *p* < 0.05—compared to the control group.

**Figure 2:**
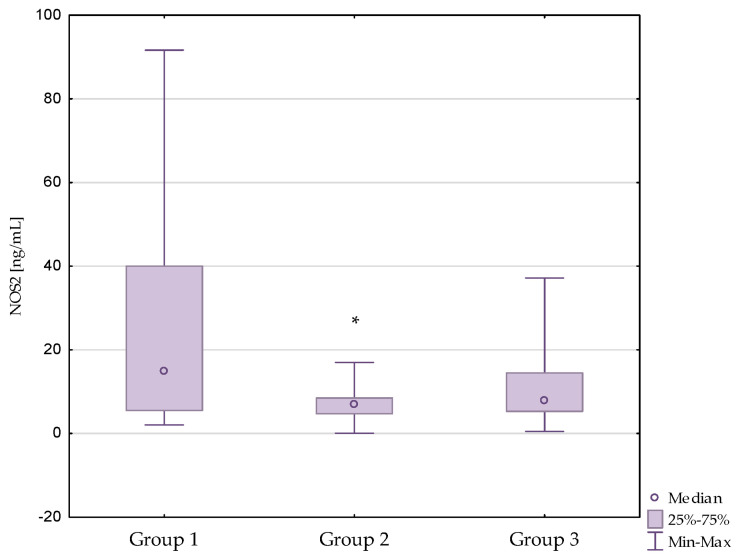
Concentrations of NOS2 in the studied groups. Group 1—control group; Group 2—diabetic nephropathy group; Group 3—kidney transplant diabetic nephropathy group. * *p* < 0.05—compared to the control group.

**Figure 3:**
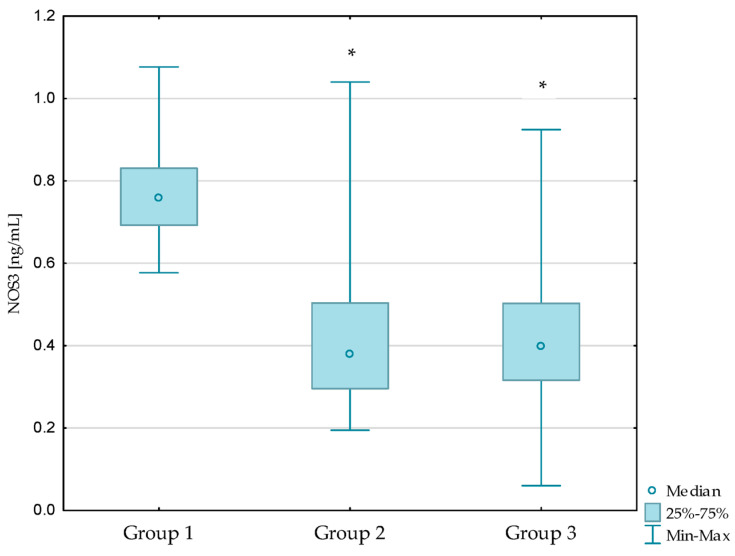
Concentrations of NOS3 in the studied groups. Group 1—control group; Group 2—diabetic nephropathy group; Group 3—kidney transplant diabetic nephropathy group. * *p* < 0.05—compared to the control group.

What is more, lower NOS3 concentrations were observed in patients with diabetic nephropathy (*p* < 0.001) and in patients with diabetic nephropathy after kidney transplantation (*p* < 0.001) compared to the control group. Similar relationships were not observed between the diabetic nephropathy group and the kidney transplant diabetic nephropathy group (*p* = 1.000). The results are presented in Figure 1, Figure 2 and Figure 3. Additionally, the results are shown in Table S1 (Supplementary Materials).

### 3.2. The Genotype Distribution of the NOS1, NOS2, and NOS3 Polymorphisms 

**Figure 4:**
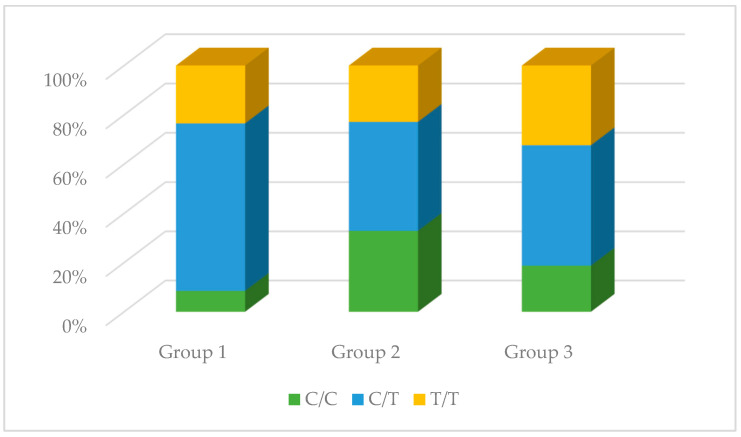
The genotype distribution of the rs3782218 polymorphism (*NOS1*) in the studied groups. Group 1—control group; Group 2—diabetic nephropathy group; Group 3—kidney transplant diabetic nephropathy group.

**Figure 5:**
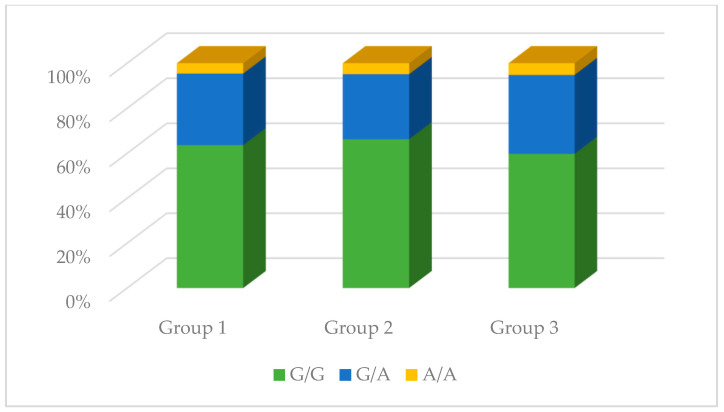
The genotype distribution of the rs1137933 polymorphism (*NOS2*) in the studied groups. Group 1—control group; Group 2—diabetic nephropathy group; Group 3—kidney transplant diabetic nephropathy group.

**Figure 6:**
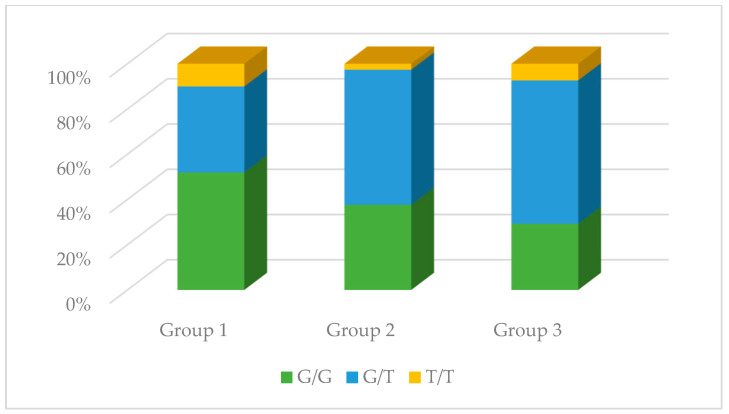
The genotype distribution of the rs1799983 polymorphism (*NOS3*) in the studied groups. Group 1—control group; Group 2—diabetic nephropathy group; Group 3—kidney transplant diabetic nephropathy group.

**Figure 7:**
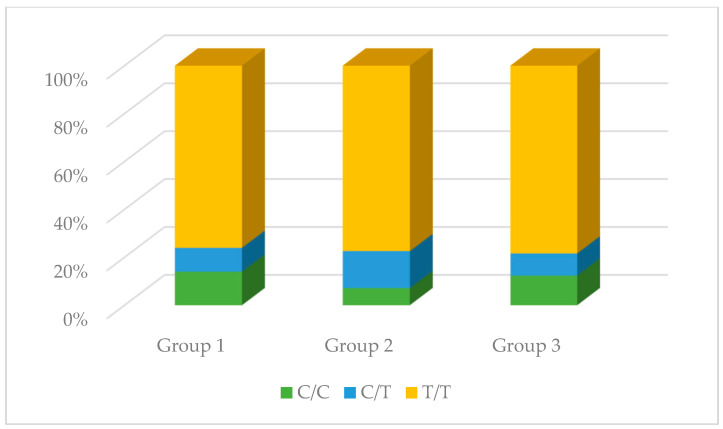
The genotype distribution of the rs2070744 polymorphism (*NOS3*) in the studied groups. Group 1—control group; Group 2—diabetic nephropathy group; Group 3—kidney transplant diabetic nephropathy group.

**Figure 8:**
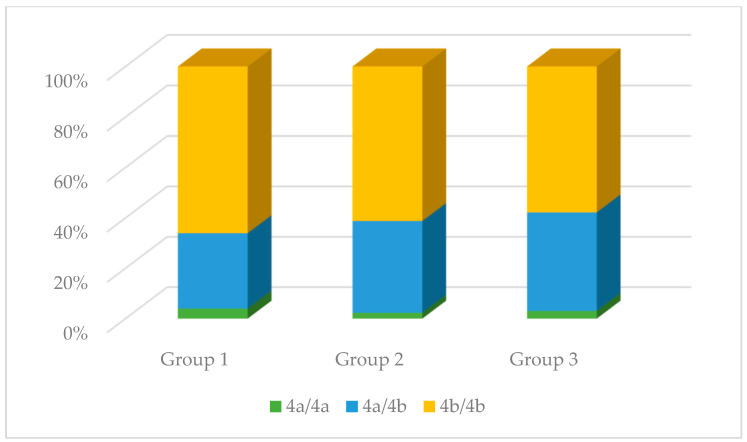
The genotype distribution of the rs61722009 polymorphism (*NOS3*) in the studied groups. Group 1—control group; Group 2—diabetic nephropathy group; Group 3—kidney transplant diabetic nephropathy group.

No significant differences were observed in the genotype distribution in the studied groups for the rs1137933 polymorphism in *NOS2* (*p* = 0.931), the rs2070744 polymorphism in *NOS3* (*p* = 0.495), and the rs61722009 polymorphism in *NOS3* (*p* = 0.839). However, in the case of the rs3782218 polymorphism in *NOS1*, these differences were present (*p* = 0.008). The C/T genotype occurred much more often compared to other genotypes in the control group (68.09%), while in the remaining studied groups, it did not constitute such an advantage. Differences in the distribution of genotypes were also observed in the case of the rs1799983 polymorphism in *NOS3* (*p* = 0.026). The G/G genotype in the control group was the dominant genotype (52.00%), while in the other two groups, the G/T genotype was dominant (59.74% and 63.41%, respectively). The results are presented in Table 2 and Figure 4, Figure 5, Figure 6, Figure 7 and Figure 8.

It was also tested whether all populations, separated according to studied groups and polymorphisms, were in Hardy–Weinberg equilibrium. Several populations were found to have significant differences between observed and expected data, indicating that these populations are not in Hardy–Weinberg equilibrium. These were the following populations: the control group with the rs3782218 polymorphism; the diabetic nephropathy group and kidney transplant diabetic nephropathy group with the rs1799983 polymorphism; and the control group, diabetic nephropathy group, and kidney transplant diabetic nephropathy group with the rs2070744 polymorphism. Due to the fact that not all populations are in the Hardy–Weinberg equilibrium, the obtained results should be approached with caution. The results of the Hardy–Weinberg equilibrium χ^2^ test are presented in Table S2 (Supplementary Materials).

### 3.3. The Influence of NOS1, NOS2, and NOS3 Polymorphisms on the Risk of Occurrence of Diabetic Nephropathy or the Likelihood of Renal Replacement Therapy

The logistic regression was used to assess the risk of developing diabetic nephropathy or the likelihood of renal replacement therapy based on *NOS1*, *NOS2,* and *NOS3* polymorphisms. It was shown that the C/C genotype of the rs3782218 polymorphism in *NOS1* is associated with a more than 12-fold increase in the odds of developing diabetic nephropathy (*p* = 0.035). However, the wide confidence interval suggests low accuracy in estimating this parameter. Additionally, it has also been observed that age may be associated with the development of this complication of diabetes. The results suggest that each subsequent year increases the chance of developing this disease by 17.90% (*p* < 0.001).

In turn, the C allele of the rs3782218 polymorphism in *NOS1* seems to be associated with an increased likelihood of renal replacement therapy (approximately 2.06-fold, *p* = 0.002). In turn, the G allele of the rs1137933 polymorphism in *NOS2* is associated with a 2.28-fold lower likelihood of renal replacement therapy (*p* = 0.007). Moreover, age may also play an important role in this case because each subsequent year increases the likelihood of renal replacement therapy by 17.70% (*p* < 0.001).

The results are presented in Table 3 and Table 4.

### 3.4. The Influence of the Studied Polymorphisms in NOS1, NOS2, and NOS3 on the Concentrations of the NOS Isoforms and Some Selected Parameters

#### 3.4.1. The Influence of the rs3782218 Polymorphism in *NOS1* on the Concentrations of the Selected Parameters

After subgrouping the population by genotype of the rs3782218 polymorphism in *NOS1*, no statistically significant differences were observed in the concentrations of NOS1 (*p* = 0.477), creatinine (*p* = 1.000), copper (*p* = 0.089), and eGFR values (*p* = 1.000).

However, higher glucose concentrations were found in the diabetic nephropathy group with the C/C genotype (*p* = 0.045), the C/T genotype (*p* < 0.001), and the T/T genotype (*p* = 0.010), as well as in the kidney transplant diabetic nephropathy group with the C/T genotype (*p* < 0.001) compared to control groups with the same genotypes.

Moreover, higher CRP concentrations were observed in the diabetic nephropathy group with the C/T genotype compared to the control group with the same genotype (*p* = 0.009), as well as in the kidney transplant diabetic nephropathy group with the C/C genotype compared to the control group with the same genotype (*p* = 0.010).

In the case of zinc concentration, lower concentrations of this metal were observed in the diabetic nephropathy group with the C/T genotype (*p* = 0.007) and in the kidney transplant diabetic nephropathy group with the C/T genotype (*p* < 0.001) compared to the control group with the same genotype.

The results are presented in Table 5.

#### 3.4.2. The Influence of the rs1137933 Polymorphism in *NOS2* on the Concentrations of the Selected Parameters

After subgrouping the population by genotype of the rs1137933 polymorphism in *NOS2*, no statistically significant differences were observed in the concentrations of NOS2 (*p* = 0.166), creatinine (*p* = 1.000), copper (*p* = 0.250), and eGFR values (*p* = 1.000).

However, it was observed that patients with diabetic nephropathy and the G/G genotype had higher glucose concentrations compared to the control group with the same genotype (*p* = 0.048). It was similar in the case of the kidney transplant diabetic nephropathy group with the G/G genotype (*p* = 0.017). Higher glucose concentrations were also observed in patients with diabetic nephropathy and the G/A genotype (*p* = 0.011) and in patients after kidney transplantation with the G/A genotype (*p* < 0.001) compared to the control group with the same genotype.

Higher CRP concentrations were also found in the diabetic nephropathy group with the G/G genotype (*p* < 0.001) and in the kidney transplant diabetic nephropathy group with the G/G genotype (*p* < 0.001) compared to the control group with the same genotype. Higher concentrations of this protein were also observed in patients after kidney transplantation with the G/A genotype compared to the control group with the same genotype (*p* = 0.003).

Lower zinc concentrations were found in the diabetic nephropathy group with the G/G genotype compared to the control group with the same genotype (*p* = 0.021) and also in the kidney transplant diabetic nephropathy group with the G/A genotype compared to the control group with the same genotype (*p* = 0.015).

The results are presented in Table 6.

#### 3.4.3. The Influence of the rs1799983 Polymorphism in *NOS3* on the Concentrations of the Selected Parameters

After subgrouping the population by genotype of the rs1799983 polymorphism in *NOS3*, no statistically significant differences were observed in the concentrations of creatinine (*p* = 1.000) and eGFR values (*p* = 1.000).

However, lower NOS3 concentrations were observed in the diabetic nephropathy group with the G/G genotype (*p* < 0.001) and in the kidney transplant diabetic nephropathy group with the G/G genotype (*p* = 0.001) compared to the control group with the same genotype. It was similar in the case of patients with the G/T genotype, where lower NOS3 concentrations were also observed in the diabetic nephropathy group (*p* < 0.001) and in the kidney transplant diabetic nephropathy group (*p* < 0.001) compared to the control group.

Higher glucose concentrations were found in the diabetic nephropathy group with the G/G genotype (*p* < 0.001) and in the kidney transplant diabetic nephropathy group with the G/G genotype (*p* < 0.001) compared to the control group with the same genotype. Higher glucose concentrations were also observed in patients with diabetic nephropathy with the G/T genotype (*p* < 0.001) and in patients after kidney transplantation with the G/T genotype (*p* < 0.001) compared to the control group with the same genotype.

In the case of CRP concentration, higher concentrations of this protein were found in patients with diabetic nephropathy and the G/G genotype compared to the control group with the same genotype (*p* = 0.011), and its higher concentrations were also observed in patients after kidney transplantation with the G/T genotype compared to the control group with the same genotype (*p* = 0.008).

Significant dependencies were also noticed in the case of the concentrations of the studied metals. Patients with diabetic nephropathy and the G/G genotype (*p* = 0.002) and patients after kidney transplantation with the G/G genotype (*p* = 0.001) had lower zinc concentrations compared to the control group with the same genotype. Also, patients after kidney transplantation with the G/T genotype had lower concentrations of this element compared to the control group with the same genotype (*p* = 0.008). Lower copper concentrations were also observed in the control group with the G/T genotype compared to the same group but with the G/G genotype (*p* = 0.027).

The results are presented in Table 7.

#### 3.4.4. The Influence of the rs2070744 Polymorphism in *NOS3* on the Concentrations of the Selected Parameters

After subgrouping the population by genotype of the rs2070744 polymorphism in *NOS3*, no statistically significant differences were observed in the concentrations of creatinine (*p* = 1.000), copper (*p* = 0.619), and eGFR values (*p* = 1.000).

Higher values of NOS3 concentration were observed in the group of patients with diabetic nephropathy and the C/C genotype (*p* = 0.049 and the T/T genotype (*p* < 0.001) and in the group of patients after kidney transplantation with the genotype T/T (*p* < 0.001) compared to control groups with the same genotypes.

It was very similar in the case of glucose, where significantly higher concentrations of this parameter were observed in the kidney transplant diabetic nephropathy group with the C/C genotype (*p* = 0.022), in the diabetic nephropathy group with the T/T genotype (*p* < 0.001) and in the kidney transplant diabetic nephropathy group with the T/T genotype (*p* < 0.001) compared to control groups with the same genotypes.

Also, higher CRP concentrations were found in the kidney transplant diabetic nephropathy group with the T/T genotype compared to the control group with the same genotype (*p* = 0.001).

Lower zinc concentrations were also observed in the diabetic nephropathy group with the T/T genotype (*p* < 0.001) and in the kidney transplant diabetic nephropathy group with the T/T genotype (*p* < 0.001) compared to the control group with the same genotype.

The results are presented in Table 8.

#### 3.4.5. The Influence of the rs61722009 Polymorphism in *NOS3* on the Concentrations of the Selected Parameters

After subgrouping the population by genotype of the rs61722009 polymorphism in *NOS3*, no statistically significant differences were observed in the concentrations of creatinine (*p* = 1.000), copper (*p* = 0.777), and eGFR values (*p* = 1.000).

Significantly lower NOS3 concentrations were observed in patients with diabetic nephropathy and the 4a/4b genotype (*p* < 0.001) and in patients after kidney transplantation and with the 4a/4b genotype (*p* < 0.001) compared to the control group with the same genotype. It was similar in the case of genotype 4b/4b, where lower NOS3 concentrations in patients with diabetic nephropathy (*p* < 0.001) were also observed and in patients after kidney transplantation (*p* < 0.001) compared to the control group with the same genotype.

Moreover, higher glucose concentrations were found in the diabetic nephropathy group with the 4a/4b genotype (*p* < 0.001) and the 4b/4b genotype (*p* < 0.001), as well as in the kidney transplant diabetic nephropathy group with the 4a/4b genotype (*p* < 0.001) and the 4b/4b genotype (*p* < 0.001) compared to the control groups with the same genotypes.

Higher CRP concentrations were also observed in the diabetic nephropathy group with the 4b/4b genotype (*p* = 0.028) and in the kidney transplant diabetic nephropathy group with the 4a/4b genotype (*p* = 0.002) and the 4b/4b genotype (*p* = 0.014) compared to the control groups with the same genotypes.

Significant differences were also noticed in the case of zinc concentration. Lower concentrations of this metal were found in the diabetic nephropathy group with the 4b/4b genotype (*p* = 0.003) and in the kidney transplant diabetic nephropathy group with the 4a/4b genotype (*p* = 0.049) and the 4b/4b genotype (*p* < 0.001) compared to the control groups with the same genotypes.

The results are presented in Table 9.

## 4. Discussion

Due to its prevalence among diabetic patients, diabetic nephropathy constitutes a significant challenge for modern medicine. It can develop for years, and despite the treatment, the patient may still need a kidney transplant in the future [36,37]. The literature has discussed the role of the *NOS* polymorphisms in the development of this disease, but the influence of the *NOS3* polymorphisms is mainly emphasized [38,39]. Therefore, the aim of this study was to investigate the impact of selected polymorphisms of all NOS isoforms on the risk of developing diabetic nephropathy or on the likelihood of renal replacement therapy. Such a comprehensive summary of the role of the *NOS* polymorphisms in the pathophysiology of diabetic complications could contribute to their better understanding and, thus, possibly the development of a faster method of diagnosing them.

The studied polymorphisms of the *NOS1* and *NOS2* (rs3782218 and rs1137933, respectively) showed no effect on the concentrations of NOS1 and NOS2. However, it was noted that individual genotypes within the *NOS3* polymorphisms (rs1799983, rs2070744, and rs61722009) were associated with differences in NOS3 concentrations between the diabetic nephropathy group and the kidney transplant diabetic nephropathy group and the control group. However, this was most likely due to the fact that respondents from the control group showed higher concentrations of this parameter compared to the other two groups (even without genotype division). Altered NO levels are often observed in patients with diabetic nephropathy [39]. There is evidence that the *NOS3* polymorphisms can influence the NO concentration, consequently leading to increased or decreased production of this compound, thereby promoting various pathological progressions [40,41]. For example, the rs1799983 polymorphism is associated with reduced NOS3 expression or activity, which corresponds well with this research. These relationships may result from the role calmodulin plays in the pathogenesis of diabetic nephropathy. Calmodulin forms the Ca^2+^/calmodulin complex, on which the activity of two NOS isoforms (NOS1, NOS3) depends [12]. Yuzawa et al. [42] proved that pancreatic β cells calmodulin-overexpressing transgenic (CaMTg) mice develop most of the changes characteristic of human diabetic nephropathy. In turn, other studies emphasized that in diabetic nephropathy there is an increase in the level of calcium–calmodulin-dependent protein kinase II (CaMKII) [43,44]. Moreover, the rs1799983 polymorphism in *NOS3* is characterized by a change of guanine (G) to thymine (T), which results in a change of glutamine (Glu) to aspartate (Asp) in the NOS3 protein. This change reduces the binding of NOS3 to caveolin-1, thereby reducing the availability of the protein for calcium-activated calmodulin activation [45,46]. This phenomenon explains the reduced activity of NOS3.

Significantly higher glucose concentrations were observed in the diabetic nephropathy group and the kidney transplant diabetic nephropathy group compared to the control group, which was a phenomenon independent of the occurring genotypes. This fact should not be surprising because these are diabetic patients with complications, and in most of them, the complications last for years [47].

This study also showed a relationship between zinc concentration and the studied *NOS* polymorphisms. However, as with NOS3 concentrations, this is most likely the result of ongoing disease and not genetic factors. Each time, these differences were concerned with lower zinc concentrations in the groups of patients with diabetic nephropathy or patients after kidney transplantation compared to the control group. Indeed, there are many examples in the literature confirming lower concentrations of this metal in patients with diabetic complications [48,49,50,51]. Zinc deficiency may be associated with inflammation or oxidative stress that accompanies many chronic diseases, including diabetes and diabetic nephropathy [48]. Al-Timimi et al. [48] indicated the role of zinc deficiency in kidney damage associated with diabetic nephropathy resulting from type 2 diabetes. The cause of this phenomenon was progressive inflammation, which was closely correlated with reduced zinc concentrations. Therefore, in patients with diabetes and diabetic nephropathy, special attention should be paid to zinc supplementation and the measurement of this parameter as part of diagnostics because long-term deficiency of this element may contribute to kidney damage [51].

However, the main aim of this study was to evaluate the selected *NOS* polymorphisms—rs3782218 (*NOS1*), rs1137933 (*NOS2*), rs1799983, rs2070744, and rs61722009 (*NOS3*)—as factors that may increase the risk of developing diabetic nephropathy or the increased likelihood of renal replacement therapy in patients with already developed diabetic nephropathy. Based on the logistic regression results, it was noticed that the rs3782218 polymorphism (*NOS1*) could potentially be used to predict the risk of developing diabetic nephropathy. The presence of the C/C genotype seems to be associated with a more than ten times greater risk of developing this complication of diabetes. This is quite interesting because most of the research related to these topics focuses on the role of the *NOS3* polymorphisms, while the rs3782218 polymorphism has not been considered an effective tool in assessing the development of this risk [52,53]. However, due to the wide confidence interval, the obtained results should be treated with caution. Moreover, it was observed that the C genotype (rs3782218, *NOS1*) is associated with an increased likelihood of renal replacement therapy, which could confirm the previously obtained result related to the C/C genotype and the development of diabetic nephropathy. In turn, the G allele (rs1137933, *NOS2*) would be associated with a reduced likelihood of this type of therapy. Unfortunately, similar information cannot be found in other studies. Perhaps this polymorphism is still understudied in the context of diabetes complications. Age also seems to be an important factor that may influence the increased risk of developing diabetic nephropathy or the increased likelihood of renal replacement therapy, which is confirmed in other studies [54,55].

## 5. Conclusions

In summary, this study indicates the potential use of the rs3782218 polymorphism (*NOS1*) in assessing the risk of diabetic nephropathy or the likelihood of renal replacement therapy. The C/C genotype and the C allele appear to be associated with an increased likelihood of diabetic nephropathy or the need for renal replacement therapy. In addition, the G allele (rs1137933, *NOS2*) appears to be associated with a reduced likelihood of renal replacement therapy. It is also confirmed that age is a factor that increases the risk of these complications. Although the role of the *NOS3* polymorphisms in the risk of developing diabetes complications has not been demonstrated, lower NOS3 concentrations were observed in patients with diabetic nephropathy or after kidney transplantation.

However, the above results should be treated as preliminary research because in order to confirm these relationships, cohort studies should be carried out, taking into account the interdependencies between these and additional factors. Nevertheless, research related to the influence of *NOS* polymorphisms on the development of diabetes complications seems to be interesting and worth continuing, as a better understanding of the pathomechanism of diabetic nephropathy may allow for its earlier diagnosis or more effective treatment.

## Figures and Tables

**Table 1:** The conditions for PCR and restriction enzyme digestion.

SNP	Primers	PCR-RFLP Conditions
rs3782218 (*NOS1*)	Forward primer—5′ CTG AGA GCA GAA GGT GGG TG 3′ Reverse primer—5′ GTC CTG GAT GGG TTT CCC TG 3′	the initial denaturation—95 °C for 15 min denaturation—95 °C for 40 s annealing—62 °C for 35 s elongation—72 °C for 40 s the final elongation—72 °C for 10 min
	**Restriction enzyme**	**Restriction enzyme digestion conditions**
	Hpy99I	37 °C for 1 h
rs1137933 (*NOS2*)	**Primers**	**PCR-RFLP Conditions**
	Forward primer—5′ CTC ACC AAA AAG TCT TCA GAC TCA CA 3′ Reverse primer—5′ GGC CCC AGT TAA ATT GTG TCT ACC 3′	the initial denaturation—95 °C for 15 min denaturation—95 °C for 40 s annealing—59 °C for 35 s elongation—72 °C for 40 s the final elongation—72 °C for 10 min
	**Restriction enzyme**	**Restriction enzyme digestion conditions**
	Hin1I	37 °C for 16 h
rs1799983 (*NOS3*)	**Primers**	**PCR-RFLP Conditions**
	Forward primer—5′ GAC CCT GGA GAT GAA GGC AG 3′ Reverse primer—5′ CAT CCC ACC CAG TCA ATC CC 3′	the initial denaturation—95 °C for 5 min denaturation—95 °C for 40 s annealing—60.4 °C for 35 s elongation—72 °C for 40 s the final elongation—72 °C for 10 min
	**Restriction enzyme**	**Restriction enzyme digestion conditions**
	MboI	37 °C for 16 h
rs2070744 (*NOS3*)	**Primers**	**PCR-RFLP Conditions**
	Forward primer—5′ CTA GTG GCC TTT CTC CAG CC 3′ Reverse primer—5′ GCC CAG CAA GGA TGT AGT GA 3′	the initial denaturation—95 °C for 15 min denaturation—95 °C for 40 s annealing—62.0 °C for 35 s elongation—72 °C for 1 min the final elongation—72 °C for 10 min
	**Restriction enzyme**	**Restriction enzyme digestion conditions**
	MspI	37 °C for 16 h
rs61722009 (*NOS3*)	**Primers**	**PCR Conditions**
	Forward primer—5′ CTA TGG TAG TGC CTT GGC TGG AG 3′ Reverse primer—5′ GTC ACA GGC GTT CCA GTA ACT AAG 3′	the initial denaturation—95 °C for 15 min denaturation—95 °C for 20 s annealing—58.0 °C for 35 s elongation—72 °C for 40 s the final elongation—72 °C for 10 min

**Table 2:** The genotype distribution of the *NOS1*, *NOS2*, and *NOS3* polymorphisms in the studied groups.

Polymorphism (*Gene*)	Groups (N)	Genotype Frequencies (%)			*p*
		C/C	C/T	T/T	
rs3782218 (*NOS1*)	Control (*N* = 47)	*N* = 4 (8.51%)	*N* = 32 (68.09%)	*N* = 11 (23.40%)	0.008
	Diabetic Nephropathy (*N* = 79)	*N* = 26 (32.91%)	*N* = 35 (44.30%)	*N* = 18 (22.79%)	
	Kidney Transplant Diabetic Nephropathy (*N* = 96)	*N* = 18 (18.75%)	*N* = 47 (48.96%)	*N* = 31 (32.29%)	
**Polymorphism (*Gene*)**	**Groups (*N*)**	**Genotype Frequencies (%)**			***p***
		**G/G**	**G/A**	**A/A**	
rs1137933 (*NOS2*)	Control (*N* = 44)	*N* = 28 (63.64%)	*N* = 14 (31.82%)	*N* = 2 (4.54%)	0.931
	Diabetic Nephropathy (*N* = 83)	*N* = 55 (66.26%)	*N* = 24 (28.92%)	*N* = 4 (4.82%)	
	Kidney Transplant Diabetic Nephropathy (*N* = 97)	*N* = 58 (59.79%)	*N* = 34 (35.05%)	*N* = 5 (5.16%)	
**Polymorphism (*Gene*)**	**Groups (*N*)**	**Genotype Frequencies (%)**			***p***
		**G/G**	**G/T**	**T/T**	
rs1799983 (*NOS3*)	Control (*N* = 50)	*N* = 26 (52.00%)	*N* = 19 (38.00%)	*N* = 5 (10.00%)	0.026
	Diabetic Nephropathy (*N* = 77)	*N* = 29 (37.66%)	*N* = 46 (59.74%)	*N* = 2 (2.60%)	
	Kidney Transplant Diabetic Nephropathy (*N* = 82)	*N* = 24 (29.27%)	*N* = 52 (63.41%)	*N* = 6 (7.32%)	
**Polymorphism (*Gene*)**	**Groups (*N*)**	**Genotype Frequencies (%)**			***p***
		**C/C**	**C/T**	**T/T**	
rs2070744 (*NOS3*)	Control (*N* = 50)	*N* = 7 (14.00%)	*N* = 5 (10.00%)	*N* = 38 (76.00%)	0.495
	Diabetic Nephropathy (*N* = 84)	*N* = 6 (7.14%)	*N* = 13 (15.48%)	*N* = 65 (77.38%)	
	Kidney Transplant Diabetic Nephropathy (*N* = 97)	*N* = 12 (12.37%)	*N* = 9 (9.28%)	*N* = 76 (78.35%)	
**Polymorphism (*Gene*)**	**Groups (*N*)**	**Genotype Frequencies (%)**			***p***
		**4a/4a**	**4a/4b**	**4b/4b**	
rs61722009 (*NOS3*)	Control (*N* = 50)	*N* = 2 (4.00%)	*N* = 15 (30.00%)	*N* = 33 (66.00%)	0.839
	Diabetic Nephropathy (*N* = 85)	*N* = 2 (2.35%)	*N* = 31 (36.47%)	*N* = 52 (61.18%)	
	Kidney Transplant Diabetic Nephropathy (*N* = 97)	*N* = 3 (3.09%)	*N* = 38 (39.18%)	*N* = 56 (57.73%)	

**Table 3:** The relationship between the selected parameters and the risk of developing diabetic nephropathy.

SNP (*Gene*)	Genotype	Diabetic Nephropathy Group	Control Group	*p*	OR	95% CI OR
rs3782218 (*NOS1*)	C/C	44	4	0.035	12.094	1.189–123.004
	C/T	82	32	0.740	0.813	0.239–2.768
	T/T	49	11	-	1.000	-
	C allele	170	40	0.300	1.275	0.850–2.019
	T allele	180	54	-	1.000	-
rs1137933 (*NOS2*)	G/G	113	28	0.893	0.897	0.183–4.385
	G/A	58	14	0.921	0.921	0.179–4.744
	A/A	9	2	-	1.000	-
	G allele	284	70	0.892	0.961	0.540–1.710
	A allele	76	18	-	1.000	-
rs1799983 (*NOS3*)	G/G	53	26	0.412	2.219	0.331–14.895
	G/T	98	19	0.273	2.809	0.444–17.777
	T/T	8	5	-	1.000	-
	G allele	204	71	0.209	0.731	0.448–1.192
	T allele	114	29	-	1.000	-
rs2070744 (*NOS3*)	C/C	18	7	0.446	0.693	0.270–1.781
	C/T	22	5	0.747	1.186	0.421–3.338
	T/T	141	38	-	1.000	-
	C allele	58	19	0.480	0.813	0.459–1.443
	T allele	304	81	-	1.000	-
rs61722009 (*NOS3*)	4a/4a	5	2	0.754	0.764	0.142–4.122
	4a/4b	69	15	0.327	1.406	0.711–2.777
	4b/4b	108	33	-	1.000	-
	4a allele	79	19	0.558	1.182	0.676–2.065
	4b allele	285	81	-	1.000	-
**Other** **Variables**	**Category**	**Diabetic** **Nephropathy Group**	**Control Group**	***p***	**OR**	**95% CI OR**
Age	-	-	-	<0.001	1.179	1.113–1.249
BMI	-	-	-	0.774	1.019	0.894–1.162
Sex	Men	94	21	-	1.000	-
	Women	90	29	0.256	0.693	0.369–1.304

These results were obtained using logistic regression analysis.

**Table 4:** The relationship between the selected parameters and the likelihood of renal replacement therapy.

SNP (*Gene*)	Genotype	Kidney Transplant Diabetic Nephropathy Group	Control Group	*p*	OR	95% CI OR
rs3782218 (*NOS1*)	C/C	18	4	0.475	1.597	0.442–5.762
	C/T	47	32	0.120	0.521	0.229–1.185
	T/T	31	11	-	1.000	-
	C allele	83	40	0.002	2.056	1.296–3.262
	T allele	109	54	-	1.000	-
rs1137933 (*NOS2*)	G/G	58	28	0.828	0.829	0.151–4.539
	G/A	34	14	0.974	0.971	0.168–5.613
	A/A	5	2	-	1.000	-
	G allele	150	70	0.007	0.438	0.242–0.795
	A allele	44	18	-	1.000	-
rs1799983 (*NOS3*)	G/G	24	26	0.531	0.767	0.334–1.761
	G/T	52	19	0.076	2.102	0.925–4.777
	T/T	6	5	-	1.000	-
	G allele	100	71	0.099	0.638	0.374–1.088
	T allele	64	29	-	1.000	-
rs2070744 (*NOS3*)	C/C	12	7	0.765	0.857	0.312–2.354
	C/T	9	5	0.856	0.900	0.282–2.872
	T/T	76	38	-	1.000	-
	C allele	33	19	0.672	0.874	0.468–1.632
	T allele	161	81	-	1.000	-
rs61722009 (*NOS3*)	4a/4a	3	2	0.896	0.884	0.140–5.567
	4a/4b	38	15	0.286	1.493	0.715–3.118
	4b/4b	56	33	-	1.000	-
	4a allele	44	19	0.467	1.251	0.685–2.283
	4b allele	150	81	-	1.000	-
**Other** **Variables**	**Category**	**Kidney Transplant** **Diabetic Nephropathy Group**	**Control Group**	***p***	**OR**	**95% CI OR**
Age	-	-	-	<0.001	1.177	1.111–1.246
BMI	-	-	-	0.785	0.982	0.864–1.117
Sex	Men	50	21	-	1.000	-
	Women	49	29	0.327	0.710	0.357–1.409

These results were obtained using logistic regression analysis.

**Table 5:** Concentrations of the selected parameters in the studied groups in terms of the rs3782218 polymorphism in *NOS1*.

Parameter	Control Group (*N* = 47)			Diabetic Nephropathy Group (*N* = 79)			Kidney Transplant Diabetic Nephropathy Group (*N* = 96)		
	C/C (*N* = 4)	C/T (*N* = 32)	T/T (*N* = 11)	C/C (*N* = 26)	C/T (*N* = 35)	T/T (*N* = 18)	C/C (*N* = 18)	C/T (*N* = 47)	T/T (*N* = 31)
NOS1 (ng/mL)	{19.47; 40.24; 48.14}	{4.41; 8.15; 25.98}	{3.86; 11.38; 24.90}	{4.40; 5.88; 16.40}	{4.07; 5.69; 11.05}	{5.30; 7.02; 13.35}	{3.93; 9.34; 22.43}	{4.12; 6.32; 15.40}	{4.92; 7.76; 21.77}
Glucose (mg/dL)	{74.43; 81.99; 90.54}	{81.99; 86.04; 88.92}	{81.00; 86.49; 93.96}	{106.00; 141.00; 183.00} *	{103.00; 124.00; 154.00} **	{115.00; 145.00; 224.00} ***	{106.50; 121.50; 149.65}	{130.00; 153.00; 213.00} **	{104.00; 132.50; 153.00}
Creatinine (mg/dL)	-	-	-	{1.20; 1.34; 1.82}	{1.12; 1.30; 1.72}	{1.04; 1.35; 1.60}	{1.03; 1.54; 2.01}	{1.14; 1.32; 1.70}	{1.12; 1.22; 1.45}
eGFR (mL/min/1.73 m^2^)	-	-	-	{30.00; 46.00; 55.00}	{36.00; 51.00; 61.00}	{39.00; 49.50; 61.00}	{35.00; 49.68; 60.50}	{40.00; 51.00; 62.00}	{51.00; 55.00; 65.50}
CRP (mg/L)	{015; 017; 0.31}	{0.34; 0.64; 0.97}	{0.50; 1.20; 1.40}	{0.24 1.54; 3.92}	{1.94; 2.38; 5.27} **	{1.03; 3.39; 4.49}	{1.49; 3.44; 8.12} *	{0.73; 1.63; 3.65}	{1.38; 2.69; 4.07}
Zn (µg/L)	{803.97; 856.71; 1101.53}	{853.50; 926.72; 1093.47}	{890.78; 964.51; 995.98}	{767.00; 835.00; 945.00}	{741.00; 826.00; 915.00} **	{739.00; 799.00; 840.00}	{632.00; 742.50; 887.00}	{733.00; 804.00; 854.00} **	{727.00; 815.00; 928.00}
Cu (µg/L)	{774.67; 827.77; 882.82}	{950.14; 995.71; 1081.33}	{1024.94; 1181.65; 1241.38}	{916.00; 1058.00; 1212.00}	{826.00; 1058.00; 1153.00}	{896.00; 1003.00; 1125.00}	{890.00; 1022.00; 1141.00}	{879.00; 1081.00; 1294.00}	{994.00; 1098.00; 1190.00}

Values are shown as {1st quartile; median; 3rd quartile}. * *p* < 0.05—compared to the control group with the C/C genotype; ** *p* < 0.05—compared to the control group with the C/T genotype; *** *p* < 0.05—compared to the control group with the T/T genotype.

**Table 6:** Concentrations of the selected parameters in the studied groups in terms of the rs1137933 polymorphism in *NOS2*.

Parameter	Control Group (*N* = 44)			Diabetic Nephropathy Group (*N* = 83)			Kidney Transplant Diabetic Nephropathy Group (*N* = 97)		
	G/G (*N* = 28)	G/A (*N* = 14)	A/A (*N* = 2)	G/G (*N* = 55)	G/A (*N* = 24)	A/A (*N* = 4)	G/G (*N* = 58)	G/A (*N* = 34)	A/A (*N* = 5)
NOS2 (ng/mL)	{6.47; 13.86; 30.92}	{4.32; 7.74; 33.37}	{40.93; 66.29; 91.66}	{5.46; 7.51; 12.11}	{4.72; 8.36; 21.64}	{1.57; 7.80; 16.41}	{5.69; 8.08; 16.42}	{4.88; 8.55; 21.79}	{9.13; 31.68; 54.22}
Glucose (mg/dL)	{80.46; 83.48; 87.48}	{84.96; 88.92; 91.08}	{75.96; 76.95; 77.94}	{101.00; 112.50; 171.50} *	{101.00; 111.50; 150.00} **	{102.00; 142.00; 228.00}	{121.00; 143.00; 173.00} *	{103.00; 139.50; 176.00} **	{110.00; 140.00; 147.00}
Creatinine (mg/dL)	-	-	-	{1.13; 1.29; 1.82}	{1.21; 1.38; 1.55}	{1.30; 1.52; 1.72}	{1.14; 1.30; 1.61}	{1.07; 1.28; 1.80}	{1.46; 1.58; 2.07}
eGFR (mL/min/1.73 m^2^)	-	-	-	{34.00; 48.00; 58.00}	{37.50; 49.50; 59.50}	{35.00; 42.00; 53.00}	{46.00; 53.00; 62.00}	{36.00; 55.50; 63.00}	{26.00; 47.00; 52.00}
CRP (mg/L)	{0.38; 0.65; 1.13}	{0.20; 0.64; 0.93}	{0.15; 0.60; 1.05}	{0.67; 3.07; 5.35} *	{0.71; 1.14; 2.38}	{1.76; 2.79; 3.14}	{0.66; 2.22; 4.70} *	{1.49; 2.87; 3.71} **	{2.66; 3.36; 3.82}
Zn (µg/L)	{843.22; 913.53; 1018.25}	{924.03; 981.37; 1031.87}	{852.79; 1077.01; 1301.23}	{752.00; 817.00; 927.00} *	{739.00; 796.00; 876.50}	{830.50; 929.50; 960.50}	{718.00; 813.50; 910.00}	{720.00; 751.00; 829.00} **	{733.00; 791.00; 902.00}
Cu (µg/L)	{866.96; 979.66; 1128.35}	{996.68; 1076.99; 1231.94}	{747.10; 1423.73; 2100.35}	{896.00; 1058.00; 1205.00}	{867.50; 1011.50; 1123.50}	{817.00; 919.00; 1009.00}	{899.00; 1080.50; 1200.00}	{935.00; 1097.50; 1254.00}	{993.00; 1025.00; 1079.00}

Values are shown as {1st quartile; median; 3rd quartile}. * *p* < 0.05—compared to the control group with the G/G genotype; ** *p* < 0.05—compared to the control group with the G/A genotype.

**Table 7:** Concentrations of the selected parameters in the studied groups in terms of the rs1799983 polymorphism in *NOS3*.

Parameter	Control Group (*N* = 50)			Diabetic Nephropathy Group (*N* = 77)			Kidney Transplant Diabetic Nephropathy Group (*N* = 82)		
	G/G (*N* = 26)	G/T (*N* = 19)	T/T (*N* = 5)	G/G (*N* = 29)	G/T (*N* = 46)	T/T (*N* = 2)	G/G (*N* = 24)	G/T (*N* = 52)	T/T (*N* = 6)
NOS3 (ng/mL)	{0.69; 0.77; 0.87}	{0.69; 0.75; 0.82}	{0.76; 0.79; 0.83}	{0.30; 0.38; 0.51} *	{0.30; 0.37; 0.49} **	{0.30; 0.67; 1.04}	{0.35; 0.43; 0.47} *	{0.30; 0.37; 0.53} **	{0.41; 0.46; 0.54}
Glucose (mg/dL)	{81.90; 85.50; 90.00}	{79.92; 84.51; 88.92}	{81.00; 86.94; 88.92}	{101.00; 135.00; 187.00} *	{101.00; 108.50; 146.00} **	{110.00; 127.50; 145.00}	{115.00; 129.00; 153.00} *	{111.50; 150.00; 173.00} **	{89.00; 134.00; 179.00}
Creatinine (mg/dL)	-	-	-	{1.07; 1.44; 1.80}	{1.16; 1.29; 1.59}	{1.60; 2.15; 2.70}	{1.14; 1.35; 2.00}	{1.07; 1.28; 1.59}	{1.44; 1.84; 2.23}
eGFR (mL/min/1.73 m^2^)	-	-	-	{34.50; 45.50; 54.50}	{36.00; 49.00; 58.00}	{24.00; 37.00; 50.00}	{36.00; 44.50; 54.00}	{45.50; 58.50; 64.00}	{25.00; 40.00; 55.00}
CRP (mg/L)	{0.34; 0.61; 1.10}	{0.15; 0.80; 1.01}	{0.53; 0.94; 1.21}	{1.94; 5.42; 7.15} *	{0.71; 1.24; 2.38}	{2.38; 2.91; 3.43}	{0.63; 2.43; 5.28}	{1.06; 2.66; 4.11} **	{0.99; 2.54; 4.09}
Zn (µg/L)	{895.37; 987.03; 1081.96}	{854.21; 964.51; 1020.85}	{803.53; 825.70; 890.78}	{744.00; 789.00; 898.00} *	{777.00; 839.00; 927.00}	{762.00; 799.00; 836.00}	{711.50; 811.00; 891.00} *	{737.50; 808.00; 918.50} **	{672.00; 730.00; 749.00}
Cu (µg/L)	{978.17; 1049.14; 1282.70}	{802.23; 912.33; 1024.94} *	{1046.20; 1100.73; 1184.39}	{930.00; 1070.00; 1205.00}	{851.00; 970.50; 1133.00}	{876.00; 1073.50; 1271.00}	{905.50; 1087.50; 1162.50}	{915.00; 1066.00; 1195.00}	{993.00; 1144.00; 1200.00}

Values are shown as {1st quartile; median; 3rd quartile}. * *p* < 0.05—compared to the control group with the G/G genotype; ** *p* < 0.05—compared to the control group with the G/T genotype.

**Table 8:** Concentrations of the selected parameters in the studied groups in terms of the rs2070744 polymorphism in *NOS3*.

Parameter	Control Group (*N* = 50)			Diabetic Nephropathy Group (*N* = 84)			Kidney Transplant Diabetic Nephropathy Group (*N* = 97)		
	C/C (*N* = 7)	C/T (*N* = 5)	T/T (*N* = 38)	C/C (*N* = 6)	C/T (*N* = 13)	T/T (*N* = 65)	C/C (*N* = 12)	C/T (*N* = 9)	T/T (*N* = 76)
NOS3 (ng/mL)	{0.76; 0.79; 0.84}	{0.69; 0.72; 0.75}	{0.69; 0.78; 0.83}	{0.30; 0.39; 0.41} *	{0.31; 0.33; 0.44}	{0.30; 0.39; 0.52} **	{0.36; 0.42; 0.75}	{0.31; 0.41; 0.50}	{0.32; 0.41; 0.54} **
Glucose (mg/dL)	{81.00; 83.52; 86.94}	{77.04; 91.98; 93.06}	{81.90; 85.50; 88.92}	{110.50; 127.50; 142.50}	{110.00; 126.00; 154.00}	{105.00; 145.00; 180.00} **	{115.00; 152.00; 183.00} *	{120.00; 146.00; 177.00}	{112.00; 136.00; 173.00} **
Creatinine (mg/dL)	-	-	-	{0.97; 1.20; 1.63}	{1.17; 1.55; 2.22}	{1.14; 1.33; 1.59}	{1.24; 1.32; 1.95}	{1.22; 1.30; 1.75}	{1.08; 1.28; 1.67}
eGFR (mL/min/1.73 m^2^)	-	-	-	{39.50; 57.00; 72.00}	{26.50; 45.50; 63.00}	{36.00; 49.00; 57.00}	{36.00; 46.00; 62.00}	{41.50; 61.00; 69.00}	{42.00; 53.50; 63.00}
CRP (mg/L)	{0.15; 0.40; 1.21}	{0.37; 0.78; 1.01}	{0.34; 0.72; 1.15}	{2.16; 2.80; 3.44}	{3.60; 3.84; 5.70}	{0.71; 1.71; 4.49}	{0.77; 3.59; 8.12}	{1.67; 2.20; 4.07}	{0.97; 2.46; 4.09} **
Zn (µg/L)	{803.53; 927.92; 995.98}	{907.67; 924.03; 1020.85}	{856.07; 972.73; 1043.34}	{788.00; 906.50; 945.00}	{759.00; 838.00; 912.00}	{744.00; 812.00; 887.00} **	{677.00; 751.00; 811.50}	{854.00; 902.00; 967.00}	{718.00; 804.50; 874.50} **
Cu (µg/L)	{868.53; 1046.20; 1100.73}	{994.74; 996.68; 1024.94}	{912.33; 998.18; 1184.39}	{961.00; 1120.50; 1205.00}	{973.00; 1145.00; 1226.00}	{851.00; 1009.00; 1129.00}	{862.50; 1053.50; 1239.50}	{1014.00; 1165.00; 1215.00}	{910.00; 1080.50; 1199.00}

Values are shown as {1st quartile; median; 3rd quartile}. * *p* < 0.05—compared to the control group with the C/C genotype; ** *p* < 0.05—compared to the control group with the T/T genotype.

**Table 9:** Concentrations of the selected parameters in the studied groups in terms of the rs61722009 polymorphism in *NOS3*.

Parameter	Control Group (*N* = 50)			Diabetic Nephropathy Group (*N* = 85)			Kidney Transplant Diabetic Nephropathy Group (*N* = 97)		
	4a/4a (*N* = 2)	4a/4b (*N* = 15)	4b/4b (*N* = 33)	4a/4a (*N* = 2)	4a/4b (*N* = 31)	4b/4b (*N* = 52)	4a/4a (*N* = 3)	4a/4b (*N* = 38)	4b/4b (*N* = 56)
NOS3 (ng/mL)	{0.61; 0.73; 0.84}	{0.69; 0.75; 0.87}	{0.70; 0.77; 0.82}	{0.27; 0.30; 0.32}	{0.26; 0.33; 0.46} *	{0.32; 0.40; 0.52} **	{0.35; 0.41; 0.47}	{0.32; 0.39; 0.45} *	{0.32; 0.44; 0.58} **
Glucose (mg/dL)	{86.04; 88.02; 90.00}	{79.92; 86.04; 93.06}	{81.00; 83.97; 88.92}	{110.00; 125.00; 140.00}	{101.00; 145.00; 191.00} *	{101.00; 107.00; 142.00} **	{90.00; 93.50; 97.00}	{115.00; 143.00; 173.00} *	{116.00; 139.00; 183.00} **
Creatinine (mg/dL)	-	-	-	{1.08; 1.17; 1.25}	{1.14; 1.45; 1.83}	{1.15; 1.32; 1.58}	{1.14; 1.19; 1.24}	{1.02; 1.16; 1.32}	{1.18; 1.41; 1.80}
eGFR (mL/min/1.73 m^2^)	-	-	-	{45.00; 48.50; 52.00}	{30.00; 45.00; 61.00}	{39.00; 50.00; 58.00}	{46.00; 60.50; 75.00}	{43.00; 59.00; 66.00}	{36.00; 51.00; 58.00}
CRP (mg/L)	{0.33; 1.16; 1.99}	{0.20; 0.64; 0.93}	{0.37; 0.78; 1.20}	{2.38; 2.91; 3.43}	{0.57; 1.03; 5.42}	{0.74; 1.91; 4.49} **	{0.77; 1.22; 1.67}	{1.49; 3.06; 5.28} *	{0.97; 2.20; 4.11} **
Zn (µg/L)	{927.92; 954.86; 981.79}	{856.07; 918.00; 1115.95}	{846.35; 964.51; 1020.85}	{788.00; 797.50; 807.00}	{737.00; 847.00; 937.00}	{755.50; 821.50; 886.50} **	{739.00; 980.00; 1015.00}	{742.00; 808.00; 921.00} *	{714.00; 787.50; 846.50} **
Cu (µg/L)	{1067.96; 1124.81; 1181.65}	{853.30; 996.68; 1232.50}	{920.91; 1018.42; 1155.12}	{1058.00; 1139.00; 1220.00}	{840.00; 1070.00; 1205.00}	{880.00; 1009.00; 1146.00}	{794.00; 1349.00; 1547.00}	{904.00; 1084.50; 1187.00}	{910.00; 1080.50; 1195.00}

Values are shown as {1st quartile; median; 3rd quartile}. * *p* < 0.05—compared to the control group with the 4a/4b genotype; ** *p* < 0.05—compared to the control group with the 4b/4b genotype.

## Data Availability

The data presented in this study are available on request from the corresponding author. The data are not publicly available due to a lack of patients’ consent to making their data public.

## Supplementary Materials

The following supporting information can be downloaded at https://www.mdpi.com/article/10.3390/antiox13070838/s1. Questionnaire S1: Sample of a questionnaire conducted among people suffering from diabetic nephropathy; Table S1: Concentrations of NOS1, NOS2, and NOS3 in the studied groups; Table S2: The Hardy–Weinberg equilibrium for studied groups; Figure S1: Example of electropherogram of the rs3782218 polymorphism in *NOS1*; Figure S2: Example of electropherogram of the rs1137933 polymorphism in *NOS2*; Figure S3: Example of electropherogram of the rs1799983 polymorphism in *NOS3*; Figure S4: Example of electropherogram of the rs2070744 polymorphism in *NOS3*; Figure S5: Example of electropherogram of the rs61722009 polymorphism in *NOS3*.
