# Supplementary material for: The Effect of Selected Nitric Oxide Synthase Polymorphisms on the Risk of Developing Diabetic Nephropathy
Source: Antioxidants (Basel). 2024 Jul 13;13(7):838. doi: 10.3390/antiox13070838 (PMC11273648; doi:10.3390/antiox13070838)
Supplement: Supplementary file 1 [file antioxidants-13-00838-s001.zip › antioxidants-3097198-supplementary.pdf]

Supplementary materials

Questionnaire S1: Sample of a questionnaire conducted among people suffering from diabetic nephropathy.

PART 1. PRELIMINARY INFORMATION

Sex [W/M]	City of residence	Age [years]	Height [cm]	Weight [kg]

QUESTION	ANSWER
Do you suffer from diabetes? If so, what type?	
Do you suffer from chronic diseases other than diabetes? If so, which ones?	
Do you suffer from diabetic nephropathy?	
Have you ever been or are you undergoing dialysis?	
Have you ever received a kidney transplant?	

PART 2. LIFESTYLE

Do you play sports? How many hours per week?	
Do you smoke cigarettes? How many years? How many packs a day?	
Do you consume alcohol? In what amount? How often?	

Do you take medications/dietary supplements? Which medications or dietary supplements?

PART 3. DISEASES IN THE FAMILY

Do your parents suffer from diabetes?	
Do your siblings suffer from diabetes?	
Do your parents suffer from diabetic nephropathy?	
Do your siblings suffer from diabetic nephropathy?	

Table S1: Concentrations of NOS1, NOS2 and NOS3 in the studied groups.

Parameter	Control Group (*N* = 50)	Diabetic Nephropathy Group (*N* = 85)	Kidney Transplant Diabetic Nephropathy Group (*N* = 97)	*p*
NOS1 [ng/mL]	{3.85; 9.35; 30.28}	{3.95; 5.30; 6.62}*	{4.22; 6.64; 15.39}	**0.002**
NOS2 [ng/mL]	{5.51; 14.82; 39.99}	{4.68; 6.87; 8.46}*	{5.28; 7.86; 14.46}	**<0.001**
NOS3 [ng/mL]	{0.69; 0.76; 0.83}	{0.30; 0.38; 0.50}*	{0.32; 0.40; 0.50}*	**<0.001**

Values are shown as {1st quartile; median; 3rd quartile}. * $p < 0.05$ – compared to the control group.

Table S2: The Hardy-Weinberg equilibrium for studied groups.

Polymorphism (*Gene*)	Groups (N)	The χ2 test values
rs3782218 (*NOS1*)	Control (*N* = 47)	**7.24**
	Diabetic Nephropathy (*N* = 79)	0.87
	Kidney Transplant Diabetic Nephropathy (*N* = 96)	<0.01
**Polymorphism (*Gene*)**	**Groups (*N*)**	**The χ2 test values**
rs1137933 (*NOS2*)	Control (*N* = 44)	0.02
	Diabetic Nephropathy (*N* = 83)	0.42
	Kidney Transplant Diabetic Nephropathy (*N* = 97)	<0.01
**Polymorphism (*Gene*)**	**Groups (*N*)**	**The χ2 test values**
rs1799983 (*NOS3*)	Control (*N* = 50)	0.30
	Diabetic Nephropathy (*N* = 77)	**10.11**
	Kidney Transplant Diabetic Nephropathy (*N* = 82)	**9.07**
**Polymorphism (*Gene*)**	**Groups (*N*)**	**The χ2 test values**
rs2070744 (*NOS3*)	Control (*N* = 50)	**22.79**
	Diabetic Nephropathy (*N* = 84)	**12.72**
	Kidney Transplant Diabetic Nephropathy (*N* = 97)	**43.72**
**Polymorphism (*Gene*)**	**Groups (*N*)**	**The χ2 test values**
rs61722009 (*NOS3*)	Control (*N* = 50)	0.03

	Diabetic Nephropathy<br>($N = 85$)	1.13
	Kidney Transplant<br>Diabetic Nephropathy<br>($N = 97$)	1.33

In order to calculate the Hardy-Weinberg equilibrium, the following values were adopted: df = 1, p = 0.05. The critical value was 3.84.

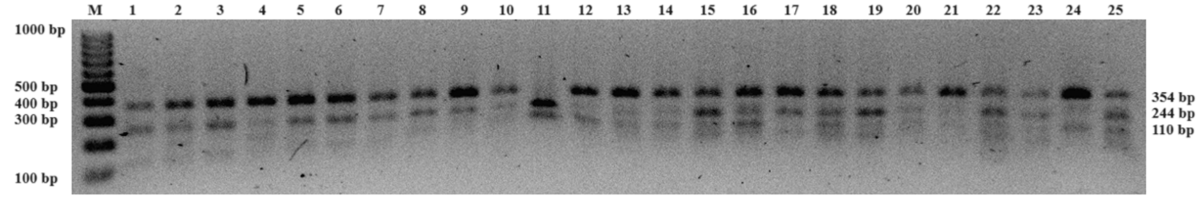

Figure S1: Example of electropherogram of the rs3782218 polymorphism in *NOS1*.
M – marker ladder; 11 – C/C genotype; 1, 2, 3, 5, 6, 7, 8, 9, 10, 12, 13, 14, 15, 16, 17, 18, 19, 20, 22, 23, 24, 25 – C/T genotype; 4, 21 – T/T genotype

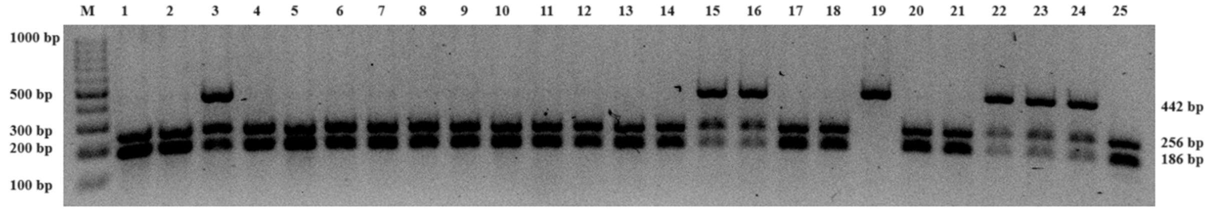

Figure S2: Example of electropherogram of the rs1137933 polymorphism in *NOS2*.
M – marker ladder; 1, 2, 4, 5, 6, 7, 8, 9, 10, 11, 12, 13, 14, 17, 18, 20, 21, 25 – G/G genotype; 3, 15, 16, 22, 23, 24 – G/A genotype; 19 – A/A genotype

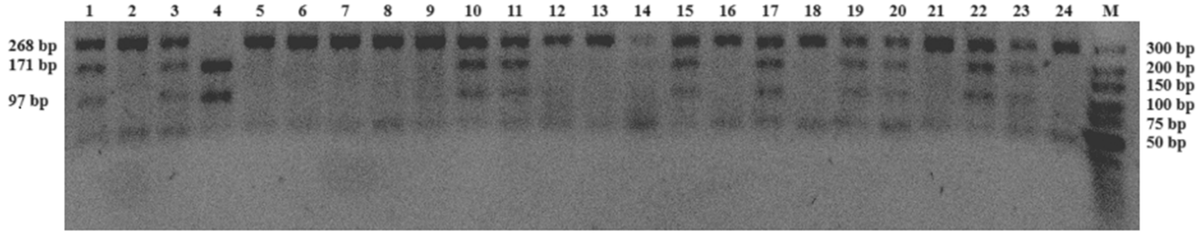

Figure S3: Example of electropherogram of the rs1799983 polymorphism in *NOS3*.
M – marker ladder; 2, 5, 6, 7, 8, 9, 12, 13, 16, 18, 21, 24 – G/G genotype; 1, 3, 10, 11, 14, 15, 17, 19, 20, 22, 23 – G/T genotype; 4 – T/T genotype

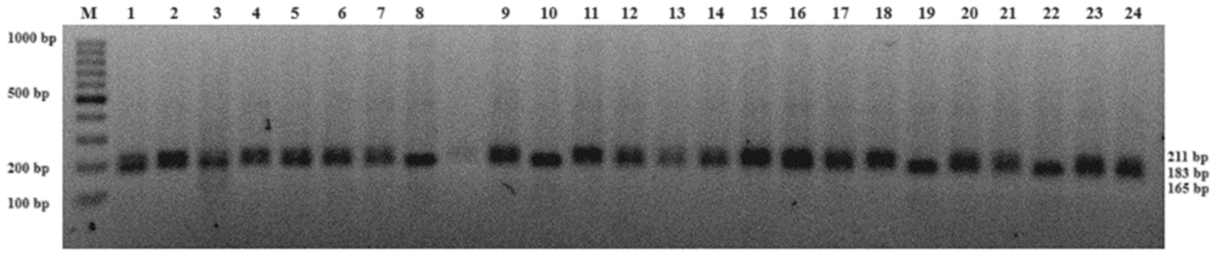

Figure S4: Example of electropherogram of the rs2070744 polymorphism in *NOS3*.
M – marker ladder; 8, 10, 19, 22 – C/C genotype; 3 – C/T genotype; 1, 2, 4, 5, 6, 7, 9, 11, 12, 13, 14, 15, 16, 17, 18, 20, 21, 23, 24 – T/T genotype

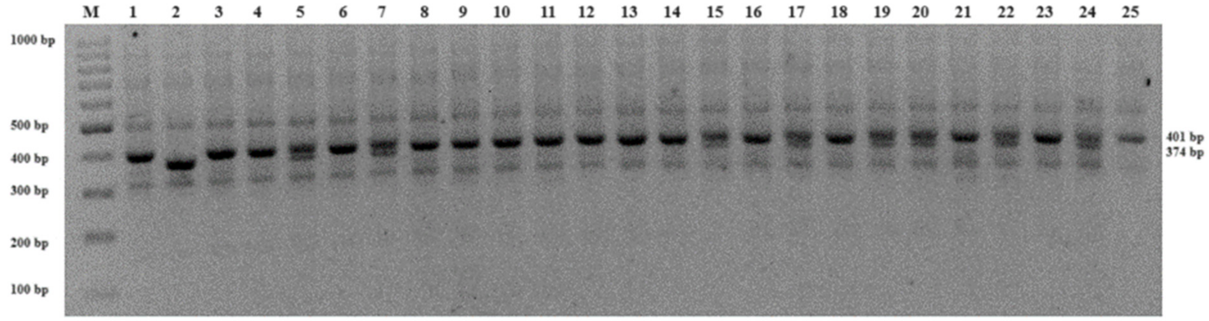

Figure S5: Example of electropherogram of the rs61722009 polymorphism in *NOS3*.
M – marker ladder; 2 – 4a/4a genotype; 5, 7, 15, 17, 19, 20, 22, 24 – 4a/4b genotype; 1, 3, 4, 6, 8, 9, 10, 11, 12, 13, 14, 16, 18, 21, 23, 25 – 4b/4b genotype
